# Skeletal muscle adaptation to microgravity: how altered mechanical cues drive catabolic signaling

**DOI:** 10.1038/s44319-026-00879-1

**Published:** 2026-07-21

**Authors:** Sandhya Singh, Samrat Chakraborty, Shenhav Shemer

**Affiliations:** Faculty of Biology, Technion Institute of Technology, Haifa, Israel

**Keywords:** Cell Adhesion, Polarity & Cytoskeleton, Musculoskeletal System, Signal Transduction

## Abstract

Exposure to microgravity induces rapid and profound skeletal muscle atrophy, yet the mechanisms by which reduced mechanical loading is sensed and translated into coordinated cellular and tissue-level responses remain incompletely understood. Accumulating evidence indicates that muscle adaptation to microgravity arises from disruption of mechanosensing pathways that regulate membrane signaling, cytoskeletal organization, and extracellular matrix (ECM) structure. Here, we integrate findings from spaceflight, bed rest, immobilization, and in vitro models to examine how reduced mechanical loading alters muscle-intrinsic signaling and tissue architecture. We discuss early suppression of anabolic pathways, activation of proteolytic systems, and remodeling of membrane-associated signaling hubs, including ECM composition, stiffness, and integrin-mediated adhesion. We further highlight how coupling between the ECM, cytoskeleton, and the nucleus enables mechanical cues to shape transcriptional programs and metabolic control during unloading. Understanding how skeletal muscle adapts to altered mechanical forces will facilitate the development of therapies for atrophy in spaceflight and terrestrial conditions of disuse, including bed rest, aging, and immobilization.

## Abstract (web summary)

Microgravity induces profound skeletal muscle atrophy with accumulating evidence pointing to disruption of mechano-sensing pathways. This review highlights signaling changes and metabolic changes, integrating findings from multiple models of muscle disuse to point toward potential therapies for muscle atrophy.

## Graphical abstract

**Figure:**
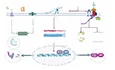

## Introduction

Gravity imposes a constant mechanical constraint that maintains skeletal muscle architecture, metabolism, and signaling. When this constraint is reduced, as during spaceflight or ground-based unloading (e.g., prolonged bed rest, limb immobilization, or inactivity during aging), the muscle rapidly atrophy through suppression of anabolic signaling and activation of proteolytic systems (Juhl et al, 2021). A major challenge has been to distinguish muscle-intrinsic responses to altered mechanical load from secondary influences such as changes in neural input, endocrine signaling, inflammation, and bone remodeling.

On unloading, early molecular events include impaired insulin/IGF-1-PI3K-AKT signaling, FOXO-dependent induction of atrogenes (e.g., MuRF1 and MAFbx/Atrogin1), and coordinated activation of the ubiquitin-proteasome system (UPS) and autophagy (Cohen et al, 2015; Lee et al, 2022). In parallel, microgravity alters membrane tension, cytoskeletal organization, and extracellular matrix (ECM) architecture (Takahashi et al, 2021; Volodin et al, 2017; Hughes-Fulford, 2003), features that are increasingly recognized as upstream modulators of growth factor responsiveness and mechanosensitive transcriptional programs.

Several recent reviews have summarized mechanotransduction pathways in skeletal muscle. Here, we integrate evidence from spaceflight studies, bed rest and immobilization in humans, hindlimb unloading in rodents, and complementary cell-based models into a unified framework for how reduced mechanical loading is sensed and translated into coordinated catabolic signaling. We emphasize three interconnected mechanisms: (i) early membrane and signaling perturbations that restrain anabolic pathways, (ii) cytoskeletal and organelle responses that amplify stress and proteolysis, and (iii) ECM-adhesion-nucleus coupling that links mechanical cues to gene expression and metabolic response. We propose that disuse atrophy emerges from a distributed mechanosensory network, rather than a single initiating sensor, in which membrane tension, adhesion complexes, cytoskeletal organization, and ion channel activity act as parallel inputs that converge on shared catabolic pathways. In this model, early suppression of anabolic signaling represents a primary response to altered mechanical input, whereas cytoskeletal remodeling and organelle dysfunction act to amplify and stabilize the atrophy state. This framework distinguishes primary mechanosensing events from downstream adaptations and highlights key unresolved questions about the hierarchy, integration, and temporal coordination of these pathways.

## Mechanical loading is a defining feature of skeletal muscle

Skeletal muscle exhibits a high degree of plasticity in response to changes in mechanical loading by adjusting its structural, contractile, and metabolic properties to functional demand. Early physiological studies have demonstrated that muscle mass, fiber-type composition, and mechanical performance are tightly coupled to loading, and reduced mechanical input consistently triggers muscle atrophy across species and experimental models (Antonutto et al, 1998; Caiozzo et al, 1994; Edgerton et al, 1995; Esser and Hardeman, 1995; Fitts et al, 2013; Gopalakrishnan et al, 2010; Vandenburgh et al, 1999). Force transmission through the sarcolemma supports anabolic signaling, maintains sarcomere organization, and preserves mitochondrial function. Conversely, reduced loading lowers the requirement for force-bearing architecture and shifts the balance toward energy conservation and protein breakdown (Baldwin et al, 1993; Parafati et al, 2025; Fitts et al, 2010; Kulesh et al, 1994; Wu et al, 2022; Ogneva et al, 2014; Widrick et al, 1999). Thus, unloading does not simply reduce contraction; it changes signaling from membrane complexes, cytoskeletal tension, and growth factor cues in muscle cells.

Muscle responses to unloading are dynamic. Although microgravity reduces mechanical load, neural and systemic inputs are partially preserved through movement and posture, whereas different ground-based unloading models remove these inputs to varying degrees, resulting in distinct muscle responses. These differences in specific mechanical and physiological context likely contribute to variability in the magnitude and kinetics of atrophy-related signaling. Thus, it is important to understand how muscles detect and transduce changes in mechanical input and how these cellular responses integrate with tissue-level adaptations to regulate muscle function under conditions of reduced loading (see below).

## Cellular responses to reduced mechanical loading

Reduced mechanical loading elicits a coordinated set of cellular responses in skeletal muscle that precede and promote loss of myofibrillar proteins. Evidence from spaceflight experiments, ground-based unloading models, and in vitro systems indicates that early suppression of insulin/IGF-1-PI3K-AKT signaling removes restraint on FOXO activity, which in turn translocates to the nucleus and activates the atrophy transcriptional program (Dupont et al, 2011; Allen et al, 2009). In parallel, unloading perturbs membrane-associated signaling hubs, such as the DGC (Murgia et al, 2022), whose association with insulin receptor and plakoglobin is important for desmin filament stability and muscle integrity (Eid Mutlak et al, 2020). Consequently, organization and integrity of the desmin cytoskeleton is reduced by unloading (Di Filippo et al, 2024), creating conditions that sensitize myofibers to catabolic cues (Volodin et al, 2017).

In addition to FOXO activation, several signaling pathways have been implicated in coordinating proteolytic responses during unloading. Activation of NF-κB signaling has been reported in rodent models of hindlimb unloading, where it contributes to the induction of muscle-specific ubiquitin ligases and promotes muscle wasting (Kandarian and Jackman, 2006). Early work demonstrated increased NF-κB DNA binding activity and transcriptional output via p50 and B cell lymphoma 3 (Bcl3) in unloaded muscle (Hunter and Kandarian, 2004), suggesting that inflammatory-like signaling can be activated in a contraction-independent manner. NF-κB and FOXO pathways may act in parallel or cooperatively, converging on shared atrophy-related gene programs.

Other pathways that are sensitive to mechanical unloading include MAPK signaling modules, particularly ERK and p38, which can be differentially regulated depending on the model and duration of unloading. For example, in rat soleus muscle, MAPK/p38 signaling is activated, and its inhibition attenuated atrophy at the early stages of muscle unloading (Belova et al, 2020). Thus, the earliest phase of disuse atrophy represents active signaling reprogramming in response to altered mechanical load, rather than a passive consequence of reduced contractile activity. Under these conditions, ERK phosphorylation was decreased, and its activation by electrical stimulation prevented slow-to-fast fiber-type transition typical of unloading without preventing soleus muscle atrophy (Dupont et al, 2011), indicating that ERK signaling alone is insufficient to counteract unloading-induced catabolism. While ERK signaling has been associated with muscle growth, its role during unloading appears complex, and its effects probably depend on integration with mechanical inputs and upstream membrane-associated signaling complexes (see below). These observations indicate that disuse atrophy is driven by a network of signaling cascades that integrate mechanical, metabolic, and stress-related inputs to regulate proteolysis.

Despite substantial progress, much of the current understanding of disuse-induced signaling remains correlative, and relatively few studies directly establish causality in vivo in the context of mechanical unloading. For example, although suppression of AKT signaling and activation of FOXO transcription factors are consistently observed in unloading models, restoring AKT activity does not fully prevent muscle loss in unloading models. These observations suggest that PI3K-AKT-FOXO signaling is necessary but not sufficient, and that multiple mechanosensitive pathways act in parallel to drive atrophy. Classical genetic studies established that activation of PI3K-AKT signaling is sufficient to override atrophy (Stitt et al, 2004; Sandri et al, 2004; Cohen et al, 2014), but these were largely performed in systemic catabolic states (e.g., fasting, glucocorticoid treatment) or transgenic models. In unloading models, experimental manipulation is more limited: for example, restoration of AKT signaling by electrostimulation during hindlimb unloading fails to prevent muscle wasting (Dupont et al, 2011), and pharmacological activation of PI3K-AKT can attenuate but not fully block atrophy (Zhang et al, 2016). Thus, while suppression of PI3K-AKT-FOXO signaling is a consistent feature of unloading, it is neither sufficient nor necessarily the primary driver of muscle loss under unloading conditions.

Similarly, alterations in cytoskeletal organization, ECM composition, and mechanosensitive channel activity are well documented, yet their relative contribution to initiating versus amplifying atrophy remains unclear (Ogneva et al, 2014; Hughes-Fulford, 2003). A key limitation is that many studies rely on in vitro systems or short-term unloading paradigms that may not fully recapitulate the complexity of muscle adaptation in vivo (Juhl et al, 2021). Moreover, differences between spaceflight, hindlimb suspension, and immobilization models complicate direct comparisons. Future studies employing muscle-specific genetic models within defined unloading paradigms, combined with time-resolved and multi-scale analyses, will be essential to distinguish primary mechanosensing events from downstream adaptive responses.

### Mechanosensitive ion channels as modulators of unloading responses

Mechanosensitive ion channels have emerged as key mediators of changes in membrane tension and intracellular signaling. Among these, members of the transient receptor potential canonical (TRPC) family and Piezo channels are particularly relevant due to their established roles in mechanotransduction in skeletal muscle (Goldermann and Hanke, 2001). TRPC channels are non-selective cation channels that localize to the sarcolemma and sarcoplasmic reticulum and contribute to calcium entry in response to membrane stretch and mechanical deformation. In skeletal muscle, TRPC-mediated calcium influx supports anabolic signaling and muscle homeostasis under normal loaded conditions (Choi et al, 2020; Franco and Lansman, 1990). Mechanical unloading, however, is consistently associated with reduced expression and activity of specific TRPC isoforms. Hindlimb suspension in rodents leads to a marked decrease in TRPC1 and TRPC3 protein levels in the soleus muscle, an effect that persists even after reloading (Zhang et al, 2014). Similarly, simulated microgravity reduces TRPC1 expression in myotubes and impairs myoblast proliferation (Benavides Damm et al, 2013), linking diminished TRPC activity to reduced calcium-dependent signaling and muscle atrophy. These findings suggest that TRPC channels do not act as primary gravity sensors but rather function as load-responsive modulators whose reduced activity contributes to unloading-induced muscle degeneration.

In contrast to TRPC channels, Piezo channels are directly gated by membrane tension and are therefore considered bona fide molecular force transducers (Syeda et al, 2016). Piezo1 and Piezo2 rapidly respond to mechanical stimuli through cation influx and activation of downstream signaling pathways that influence cell growth, differentiation, and metabolism (Mirzoev, 2023). Experimental evidence indicates that Piezo channel gating can occur independently of cytoskeletal attachments (Cox et al, 2016), although interactions with ECM components modulate their sensitivity to applied forces (Gaub and Müller, 2017). In skeletal muscle, reduced mechanical loading due to denervation, immobilization, or disuse is associated with decreased Piezo1 expression, concomitant induction of the transcription factor KLF15, and induction of atrophy-related genes (Bodine et al, 2001; Mirzoev, 2023). A key question is how channel-dependent calcium signals integrate with membrane scaffolds, cytoskeletal tension, and growth factor signaling during unloading. In this context, mechanosensitive channels may regulate the threshold and timing of catabolic activation by influencing the activity of calcium-dependent proteases (Cohen, 2020), mitochondrial stress signaling, and transcriptional programs. Clarifying when and where these channels operate in vivo, relative to early AKT suppression and membrane complex remodeling, will be essential for defining their contribution to microgravity-induced muscle loss.

While calcium influx represents a central output of mechanosensitive channel activation, emerging evidence suggests that these channels also regulate broader signaling networks. For example, Piezo1 activation has been linked to the regulation of transcriptional programs through YAP/TAZ signaling in bone, where it modulates osteoclastogenesis (Wang et al, 2020). Conversely, Piezo1 deficiency suppresses this pathway and promotes bone resorption, suggesting that impaired mechanosensitive signaling can drive catabolic tissue remodeling. Whether a similar Piezo1/YAP/TAZ mechanism operates in skeletal muscle, and how it might influence muscle mass under unloading, remains largely unknown. In addition, ion flux through these channels can influence membrane potential, mitochondrial function, and reactive oxygen species production, thereby indirectly shaping metabolic and stress signaling.

Mechanosensitive channels may also interface with kinase signaling pathways, including MAPKs (Martineau and Gardiner, 2001) and potentially NF-κB (Kumar and Boriek, 2003), although these connections remain incompletely understood in skeletal muscle. Importantly, changes in channel activity during unloading are likely to affect not only calcium-dependent proteases such as calpains but also transcriptional and metabolic responses that precede overt protein degradation (Cohen, 2020; Aweida et al, 2018). Mechanosensitive ion channels therefore function as integrators of mechanical and biochemical signals that regulate transcription, metabolism, and stress responses during unloading. Despite these advances in the roles of mechanosensitive ion channels, most evidence remains correlative, and their precise contribution to initiating versus amplifying atrophy signaling in vivo is still unresolved.

### Cytoskeletal remodeling under unloading conditions

The cytoskeleton plays a central role in translating mechanical forces into intracellular responses and is profoundly affected by reduced loading. In skeletal muscle, cytoskeletal elements, including actin filaments, microtubules, and intermediate filaments such as desmin, form an integrated network that links the contractile apparatus to the sarcolemma, ECM, and intracellular organelles (Agnetti et al, 2021). This network maintains force balance and enables efficient force transmission under physiological loading. Mechanical unloading disrupts this equilibrium, leading to extensive cytoskeletal reorganization (Hughes-Fulford, 2003). Spaceflight and unloading studies in animals and cultured cells consistently report alterations in cytoskeletal architecture, changes in cell shape, and impaired alignment of myofibrils (Wu et al, 2022; Fitts et al, 2010; Kulesh et al, 1994; Ogneva et al, 2014; Widrick et al, 1999). Even brief exposure to microgravity can perturb cytoskeletal organization (Widrick et al, 1999), suggesting that these structures are highly sensitive to changes in mechanical strain. These structural changes were associated with reduced sensitivity to calcium and increased shortening velocity, suggesting altered myofilament lattice spacing. Similar effects, though more severe, were observed in human muscle biopsies following prolonged spaceflight (180 days) and in bedridden patients (Fitts et al, 2010, 2013). Such remodeling has direct functional consequences, as disruption of the desmin cytoskeleton alone is sufficient to impair force transmission and compromise muscle integrity (Volodin et al, 2017; Agnetti et al, 2021). Cytoskeletal reorganization under unloading conditions also affects myoblast fusion and myotube formation (Kulesh et al, 1994). Proper fusion requires aligned cytoskeletal structures and mechanical tension, both of which are disrupted under microgravity or simulated unloading. Impaired fusion contributes to altered myofiber architecture and may exacerbate muscle weakness during prolonged unloading. In addition, reduced mechanical resistance lowers cytoskeletal tension and can destabilize membrane–cytoskeleton associations that normally support costamere organization and responsiveness to growth factors. Such changes are not merely structural; they influence the distribution and activity of signaling nodes that regulate protein synthesis, proteolysis, and mitochondrial function.

Cytoskeletal remodeling can intersect with canonical atrophy pathways. Changes in actin and/or desmin filament dynamics and membrane anchoring can influence mechanosensitive transcriptional regulators and modulate signaling via nitric oxide synthase (nNOS) (Gonzalez et al, 2015) and insulin-PI3K-AKT signaling (Eid Mutlak et al, 2020; Murgia et al, 2022). Altered cytoskeletal tension and organization can also influence focal adhesion complexes and associated kinases, including focal adhesion kinase (FAK) (Durieux et al, 2007), change the accessibility of myofibrillar substrates to proteolytic systems (Cohen et al, 2012; Volodin et al, 2017), and amplify stress signaling through mitochondrial and calcium-dependent modules. These observations place cytoskeletal perturbations upstream of, and synergistic with, the proteolytic programs (e.g., UPS, autophagy, calpains) that drive myofibril loss and atrophy during unloading.

### Organelle-based responses to reduced mechanical input

Mechanical unloading rapidly impacts organelle homeostasis, particularly mitochondria and the sarcoplasmic reticulum. Reduced loading is associated with changes in mitochondrial morphology, distribution, and redox balance (Serano et al, 2025; Mirzoev et al, 2021), that parallel those observed during disuse and aging. Whether organelle remodeling contributes directly to mechanosensing or reflects downstream metabolic adaptation remains an open question (see Box 1). Reduced mitochondrial function may amplify unloading-induced muscle loss by impairing energy supply and anabolic signaling and promoting oxidative stress and catabolic signaling. Similarly, alterations in sarcoplasmic reticulum function and calcium handling under unloading conditions can disrupt excitation–contraction coupling and gene expression (Khan et al, 2022), further contributing to muscle dysfunction. Clearly, mitochondrial dysfunction is not an isolated consequence of atrophy but part of an interconnected response to unloading that includes alterations in mechanosensitive ion channels, cytoskeletal networks, and organelle adaptations. All converge to modulate calcium signaling, protein turnover, and metabolic homeostasis, leading to functional decline. The relative contributions of these mechanisms to atrophy likely depend on muscle activity, loading history, and physiological context. An intriguing question is how these cellular processes are coordinated at the tissue level through ECM interactions and muscle fiber architecture.

Box 1 In need of answers

- **What are the earliest molecular events that initiate disuse atrophy?**
  Identifying primary mechanosensing events will require time-resolved in vivo studies that capture early signaling changes immediately following unloading.
- **Do mechanosensory systems act hierarchically or in parallel?**
  Dissecting the relative contributions of adhesion complexes, ion channels, and cytoskeletal networks will require selective perturbation of each system within the same experimental framework.
- **How is mechanical information integrated at the sarcolemma?**
  Defining how membrane tension, receptor signaling, and ion flux are coordinated will require high-resolution imaging and quantitative measurements of membrane-associated signaling complexes.
- **What determines the balance between anabolic suppression and catabolic activation?**
  Understanding how reduced insulin/IGF-1 signaling interfaces with stress-responsive pathways during unloading will require combined genetic and biochemical approaches in defined unloading models.
- **How can muscle-intrinsic mechanosensing be isolated from circulating and neural influences?**
  Dissecting intrinsic responses to mechanical unloading will require in vitro systems that simulate microgravity (e.g., Random Positioning Machine) or reduced mechanical load in the absence of systemic inputs, such as advanced culture platforms and random positioning-based approaches that enable controlled unloading of muscle cells.
- **To what extent are disuse-induced signaling pathways reversible?**
  Determining whether mechanosensitive signaling can be restored independently of mechanical load will be critical for developing therapeutic strategies. For example, muscle atrophy in astronauts due to exposure to microgravity in short-term spaceflight expeditions is mostly reversible; however, long-term space missions can cause irreversible effects. Besides in-flight exercise countermeasures, other approaches such as neuromuscular electrical stimulation and inducing torpor in astronauts can be effective countermeasures to attenuate muscle wasting.
- **Are mechanisms of disuse sensing conserved across models?**
  Resolving differences between spaceflight, hindlimb unloading, and cast immobilization will require systematic comparative studies across species and experimental systems.

## ECM–integrin–nucleus signaling and tissue integration

Mechanical inputs are sensed not only by the contractile apparatus but also at the muscle tissue level, where the ECM and associated adhesion complexes couple tissue-level forces to intracellular signaling and gene expression programs in the nucleus (Roberts, 2016). In skeletal muscle, integrin-based costameres and the dystrophin–glycoprotein complex (DGC) connect the ECM to the cytoskeleton and, through interconnected cytoskeletal networks, to the nucleus. Under normal loading, these assemblies stabilize sarcolemmal integrity, coordinate force transmission, and support anabolic signaling. When mechanical input is reduced, as in microgravity or prolonged unloading, remodeling of the ECM and its adhesion complexes can lower mechano-sensation by muscle, weakening response to growth factors, and thus favoring the activation of catabolic transcriptional programs (Bradbury et al, 2020; Louis et al, 2015).

### Remodeling of ECM composition under unloading

Microgravity and ground-based unloading models indicate early changes in ECM turnover, although the magnitude can vary with experimental model, duration of unloading, and readout (whole muscle *vs*. isolated fibers). Across paradigms, three common features emerge: (i) altered abundance of ECM structural proteins and basement-membrane components (Murgia et al, 2022; Urso, 2009), (ii) changes in collagen organization and crosslinking (Kjaer, 2004), and (iii) remodeling of ECM-associated proteases and their inhibitors (Carmeli et al, 2004). Even modest changes in ECM composition or stiffness can alter integrin engagement and downstream signaling in muscle fibers (Boppart and Mahmassani, 2019; Hassan et al, 2019).

### Altered ECM stiffness influences cellular signaling under muscle unloading

Beyond composition, unloading is expected to change the mechanical properties of the muscle's extracellular environment, particularly tissue stiffness. Importantly, stiffness is unlikely to change uniformly across different disuse conditions. Depending on duration, muscle type, and the balance between matrix deposition and degradation, unloading may lead to either local softening (loss of organized matrix/tension) or relative stiffening (maladaptive matrix remodeling and crosslinking). In both cases, deviations from the physiological stiffness range are likely to disrupt mechanosensitive signaling in muscle fibers.

Skeletal muscle cells function in response to mechanical cues, and integrin-based adhesions are sensitive to changes in substrate stiffness. Even modest shifts in ECM mechanical properties can alter integrin engagement, adhesion maturation, and downstream signaling through FAK and associated pathways (Sun et al, 2016). Stiffness-dependent signaling intersects with metabolic control. For example, reduced mechanical resistance can lower cytoskeletal tension, destabilize membrane-associated signaling hubs, and impair insulin-PI3K-AKT signaling, thereby favoring FOXO activation and catabolic gene expression. At the tissue level, stiffness heterogeneity due to unloading may further exacerbate dysfunction by disrupting coordinated force transmission across fibers and between fibers and their niche (Ogneva, 2010). Such mechanical mismatches could amplify local stress, promote focal signaling defects, and impair the integration of myofibers within the surrounding matrix. Thus, unloading-induced changes in ECM stiffness may represent a critical layer of regulation linking matrix remodeling to muscle cell signaling and atrophy.

### Integrin-based costameres as mechanosensory hubs

Integrins (e.g., α7β1 in adult skeletal muscle) organize costamere platforms that link mechanical cues with signaling. Engagement of integrins activates talin, kindlin and associated proteins including vinculin to activate signaling through FAK/Src kinases, which can intersect with PI3K-AKT-mTOR signaling and small GTPases (Zhao et al, 2018). Consequently, mechanical inputs appear to activate pathways controlling protein synthesis, cytoskeletal dynamics, and survival. Under unloading, reduced strain is expected to lower integrin tension and shift the balance from stable, force-bearing adhesions toward more labile assemblies. In cell culture systems, reduced adhesion signaling often correlates with diminished FAK activity and the downstream PI3K-AKT signaling, which in muscle should drive atrophy (Latres et al, 2005; Durieux et al, 2007; Zhao et al, 2018). Thus, loss of integrin-FAK signaling provides a possible mechanism by which ECM changes and reduced external force are translated into the intracellular catabolic state that characterizes disuse. In addition, integrin signaling may influence calcium handling and reactive oxygen species production indirectly through cytoskeletal and mitochondrial organization. Because mitochondrial distribution and function are sensitive to cytoskeletal architecture, and because costameres coordinate cortical actin and desmin networks, integrin remodeling can propagate to metabolic modules that amplify stress signaling during microgravity exposure.

### The DGC contributes to integrin signaling

The DGC contributes to integrin adhesions by stabilizing the sarcolemma and enabling lateral force transmission by linking the ECM to the cytoskeleton. Beyond its structural role, the DGC functions as a signaling hub that regulates pathways controlling muscle mass, including signaling via nNOS signaling (Gonzalez et al, 2015) and insulin receptor activity (Eid Mutlak et al, 2020; Murgia et al, 2022; Carney et al, 2023). Reduced DGC integrity impairs these signaling pathways and can sensitize muscle fibers to catabolic activation (Acharyya et al, 2007; Shemer, 2026; Araujo et al, 2013), even in the absence of overt mechanical injury (Gorza et al, 2021). Evidence from mechanically stimulated tissues supports the concept that force is transduced into intracellular signaling through membrane-associated complexes and ion channels. Mechanical stretch activates NF-κB and AP-1 transcription factors via pathways involving stretch-activated ion channels, reactive oxygen species, and MAPK signaling, including ERK1/2 and p38 (Kumar et al, 2003). In muscle, mechanical stress activates MAPK pathways in a manner that depends on the direction of force application, with distinct upstream signaling requirements for axial versus transverse loading (Kumar et al, 2002), indicating that mechanotransduction is not only force-dependent but also spatially encoded. These findings highlight that mechanical inputs are interpreted through structured membrane-cytoskeleton systems rather than through uniform signaling outputs. Importantly, disruption of DGC integrity alters these mechanotransduction pathways. In dystrophin-deficient *mdx* mouse muscle, mechanical stretch induces exaggerated ERK1/2 activation and enhanced AP-1 transcriptional activity, accompanied by altered calcium dependence and increased reliance on stretch-activated channels (Kumar et al, 2004). These changes indicate that dystrophin is not only a load-bearing element but also a regulator of how mechanical inputs are translated into intracellular signaling. Loss of DGC integrity therefore leads to aberrant mechanotransduction, characterized by MAPK activation, altered calcium signaling, and increased activation of stress-responsive transcriptional programs.

These observations also shed light on the complex role of ERK signaling in the regulation of muscle mass. While ERK activation can support muscle growth and regeneration downstream of mechanical loading and growth factor signaling, it fails to prevent atrophy during unloading (Dupont et al, 2011). Instead, ERK function likely depends on integration with membrane-associated complexes such as the DGC and integrin adhesions, which define the balance between anabolic and stress-activated outputs. Given that unloading reduces the need for mechanical resistance, microgravity may preferentially reveal the signaling functions of these complexes. When there is less mechanical force, maintenance and turnover mechanisms may promote partial disassembly of costameres and DGC components, leading to impaired insulin/IGF-1-PI3K-AKT signaling and reduced anabolic responsiveness. At the same time, altered membrane organization and ion channel activity may shift signaling toward MAPK-, NF-κB-, and AP-1-dependent pathways, thereby promoting catabolic transcriptional programs. These findings support a model in which the DGC and associated adhesion complexes act as signaling filters that determine how mechanical inputs are interpreted at the sarcolemma, coupling structural integrity to the regulation of muscle mass.

## Cytoskeletal coupling to the nucleus

Mechanical forces and cytoskeletal tension are transmitted to the nucleus through the linker of nucleoskeleton and cytoskeleton (LINC) complex, which connects actin, intermediate filaments, and microtubules to the nuclear lamina (Hieda, 2019). This coupling influences nuclear positioning, chromatin organization, and transcriptional output. In muscle, where nuclei are spatially organized along the muscle fiber, maintaining nuclear architecture is crucial for coordinated gene expression and for the nuclear microenvironment. On unloading, reduced cytoskeletal tension is expected to shift the nuclear mechanics toward a low-tension state that favors catabolic and stress-responsive transcriptional profiles (Cho et al, 2017). Potential consequences include changes in chromatin accessibility, altered activity of mechanosensitive transcriptional regulators, and consequently reprogramming of gene expression networks. Mechanosensitive regulators such as YAP/TAZ and SRF–MRTF pathways, both influenced by actin dynamics and adhesion tension, are possible mediators linking costamere remodeling to transcriptional changes (Fearing et al, 2019). Under loading, these pathways typically support cytoskeletal gene expression and cell growth (Mason et al, 2019; Speight et al, 2016). Reduced β-actin or desmin tension may drive their inactivation, reducing structural integrity and contributing to atrophy.

A key limitation of in vitro models is that muscle atrophy is a tissue-level process influenced by interactions among myofibers, fibroblasts, endothelial cells, immune cells, and the ECM. Microgravity therefore influences not only myofiber-intrinsic pathways but also the behavior of non-muscle cells within the matrix niche. Changes in fibroblast activity can result in aberrant formation of ECM and structural instability (Mayorca-Guiliani et al, 2025), which in turn alter myofiber mechanosensing and regeneration capacity. These findings suggest that ECM-integrin-nucleus coupling functions as a central integrative pathway at the tissue level, linking ECM remodeling to altered signaling and transcriptional responses in muscle.

## Conclusions

**Figure 1:**
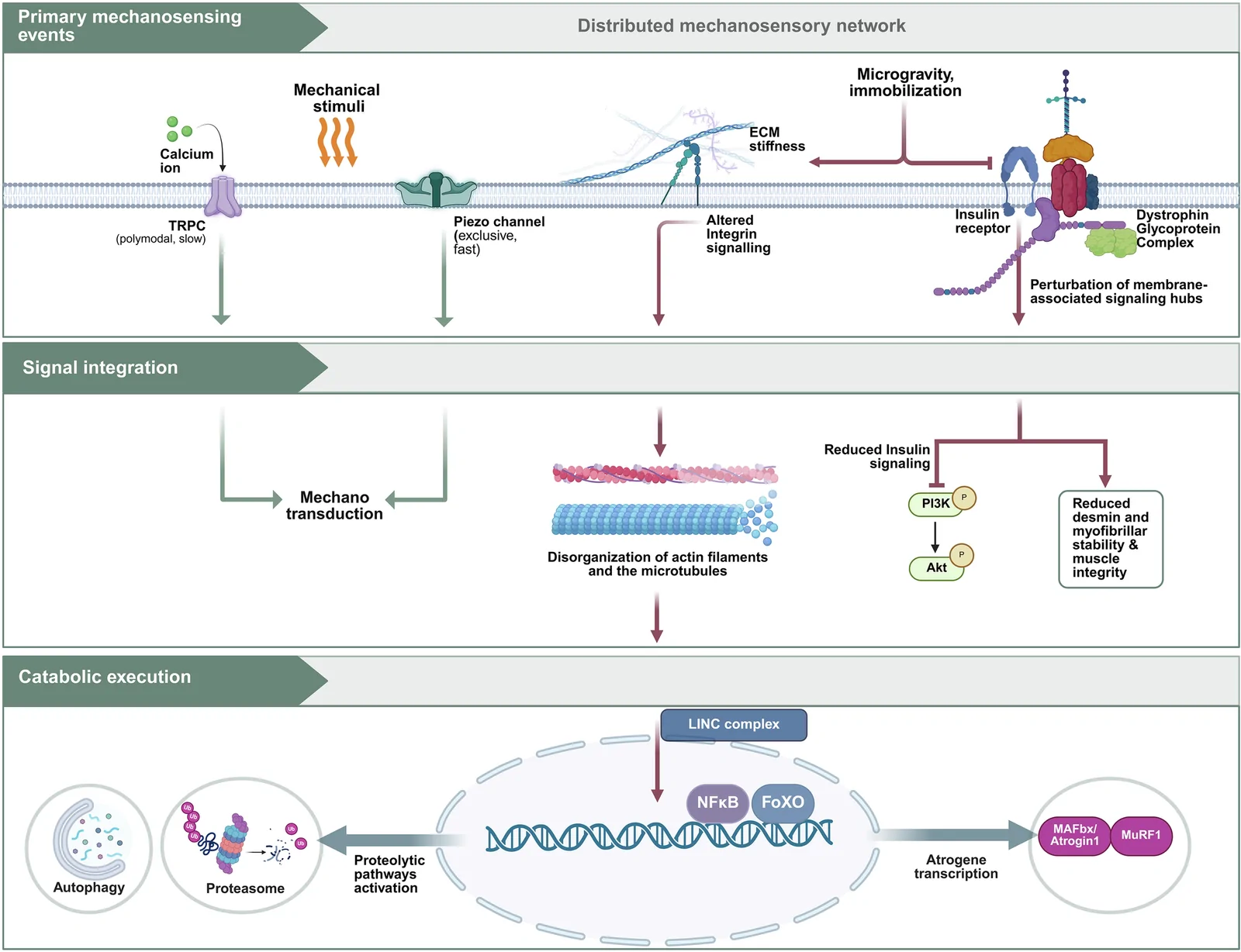
Distributed mechanosensing under unloading drives coordinated catabolic signaling in skeletal muscle. Reduced mechanical loading, as occurs during microgravity or ground-based unloading models (e.g., hindlimb suspension), alters membrane tension and adhesion-dependent signaling in skeletal muscle fibers. At the sarcolemma, mechanosensory inputs, including integrin-based adhesions, the DGC, and mechanosensitive ion channels (including TRPC and Piezo channels), detect changes in mechanical load and suppress insulin/IGF-1-PI3K-AKT signaling. In parallel, altered ion channel activity and cytoskeletal remodeling amplify stress signaling and destabilize intracellular organization. Mechanical information is transmitted to the nucleus via cytoskeletal networks and the LINC complex, leading to activation of transcriptional regulators such as FOXO and NF-kB, which induce proteolytic pathways, including the UPS (including the major atrogenes MuRF1 and MAFbx/Atrogin1) and autophagy.

A central challenge in the field of disuse atrophy has been to define how reduced mechanical loading is initially sensed and how this signal is propagated to drive coordinated catabolic responses. Based on current evidence, we propose a model in which disuse is detected through a mechanosensory network (Hughes-Fulford, 2003; Hassan et al, 2019; Palmisano et al, 2015) in which early changes in membrane tension and adhesion signaling reduce the activity of anabolic pathways, particularly insulin-PI3K-AKT signaling (Zaripova et al, 2025; Zhang et al, 2016; Dupont et al, 2011). In parallel, cytoskeletal remodeling and altered ion channel activity amplify stress signals and destabilize intracellular organization (Ogneva et al, 2014; Mirzoev, 2023). These events converge on transcriptional regulators, including FOXO and NF-κB, to activate proteolytic programs (Cai et al, 2004; Sandri et al, 2004) (Fig. 1). This model distinguishes between primary sensing events (e.g., altered membrane tension, adhesion signaling) and secondary adaptations (e.g., mitochondrial dysfunction, ECM remodeling), while acknowledging that these processes rapidly become interconnected. Such a distributed sensing mechanism may explain why multiple perturbations, including mechanical, metabolic, and structural, can independently trigger similar atrophy programs.

Mechanosensitive ion channels (e.g., TRPC, Piezo1), cytoskeletal networks, ECM-integrin-nucleus connections, and organelle-based responses contribute to sensing and transducing altered mechanical cues. At higher levels of organization, these cellular events are integrated with fiber-type composition, sarcomere architecture, muscle stiffness, and activity-dependent inputs. The relative contribution of each mechanism likely depends on the mechanical history of the muscle, the degree of unloading, and the presence of compensatory activity such as exercise. Thus, effective therapeutic strategies may need to preserve not only contractile activity but also ECM-adhesion integrity and mechano-genomic coupling. Defining how these pathways interact across time and across muscle groups will be critical for improving interventions in spaceflight and in terrestrial conditions of disuse.

A key challenge is to distinguish primary mechanosensitive events from secondary adaptations arising from reduced contractile activity or altered metabolic demand. Addressing this will require time-resolved in vivo studies that directly test causality within defined unloading paradigms (see Box 1). Understanding how mechanosensory pathways are integrated across molecular, cellular, and tissue levels will be essential for developing strategies to preserve muscle mass in conditions where mechanical loading cannot be fully restored, both in spaceflight and in terrestrial disease.

### Graphics

The figure was created with BioRender.com.

## Supplementary information

Peer Review File
