# Supplementary material for: Skeletal muscle adaptation to microgravity: how altered mechanical cues drive catabolic signaling
Source: EMBO Rep. 2026 Jul 21;27(16):4614–23. doi: 10.1038/s44319-026-00879-1 (PMC13503944; doi:10.1038/s44319-026-00879-1)
Supplement: Supplementary file 1 [file 44319_2026_879_MOESM1_ESM.pdf]

EMBO reports

Skeletal muscle adaptation to microgravity: how altered mechanical cues drive catabolic signaling

Sandhya Singh, Samrat Chakraborty, and Shenhav Shemer

Corresponding author(s): Shenhav Shemer (shenhavc@technion.ac.il)

Review Timeline:

Submission Date:	23rd Jan 26
Editorial Decision:	30th Mar 26
Revision Received:	23rd Apr 26
Accepted:	23rd Jun 26

Editor: Kurt Weir

Transaction Report:

(Note: With the exception of the correction of typographical or spelling errors that could be a source of ambiguity, letters and reports are not edited. Depending on transfer agreements, referee reports obtained elsewhere may or may not be included in this compilation. Referee reports are anonymous unless the Referee chooses to sign their reports.)

1st Editorial Decision **30th Mar 2026**

Dear Prof. Shemer,

Thank you once more for the submission of your review to EMBO Reports. I have now received two referee reports that are copied below. As you will see, both referees state that your manuscript is interesting and timely, but they have also several suggestions to improve it which I kindly ask you to address, particularly delving into more recent literature and highlighting unresolved questions.

Please also address the following editorial points:
- When returning a revised version, may I also kindly ask you to include a box called "In need of answers" that briefly outlines the major questions that are still open in a given field in the form of a few bullet points. These questions can be accompanied by a brief explanation of what would be needed to address them and may prove helpful towards setting the stage for future experimentation in the field.

- Please limit the number of keywords on the title page to five.
- Please rename the 'Competing Interests' section to 'Disclosure and competing interests statement'. For more information see https://www.embopress.org/page/journal/14693178/authorguide#conflictsofinterest
- As we are switching from a free-text author contribution statement towards a more formal statement based on Contributor Role Taxonomy (CRediT) terms, please remove the present Author Contribution section and instead specify each author's contribution(s) directly in the Author Information page of our submission system during upload of the final manuscript. See https://casrai.org/credit/ for more information.

- When submitting your revised manuscript, we will require a Microsoft Word file (.docx) of the revised manuscript text including detailed figure legends (at the very end), but without the figures.
- Please provide the final figures as separate, high resolution files as .pdf, .eps, .tif, or .jpg (one file per figure). Please finalize the drafts provided and make sure they accurately illustrate the key scientific concepts that you wish to show.

Please also note the following general advice on review figures:
- If there are certain aspects of your figure draft that are based upon assumptions or where the scientific data remains ambiguous (for example, schematically depicting a presumed direct protein-protein interaction, protein shape or subcellular localizations etc.) please add a comment so that we can work with you on an accurate depiction. Please ensure the directionality and nature of interactions is presented accurately.
- If the figure or single panels of the figure have been adapted from a published figure, please add this information to the figure legend (e.g., 'Adapted from...' or 'Based on...'). The editor will discuss if a reference and permission will be necessary
- Please only re-use figures or parts of a figure if this is essential for understanding the concept communicated. Often a reference to a previous paper will suffice. If the figure contains re-used images or elements of images, including schematics, micrographs or photos, please make sure that you have the permission/license to publish it (this also applies to your own previous work, if the journal you published in retains copyright. Certain 'creative commons' open access licenses, such as CC-BY 4.0, allow re-use without additional formal permissions). All re-used material must be explicitly cited.
- If you use an image data base for scientific iconography (e.g., BioRender), please let us know if you have a license that allows for publication in an academic journal. Often authors use misleading iconography for expedience. Please ensure the information shown is scientifically accurate. If in doubt, please discuss with the editor.

Thank you again for writing this article for us. I look forward to seeing a revised version of your manuscript when it is ready. As for timing, would it be possible for you to submit the revised version by end of June? But let me know if you need more time.

Best regards,

Kurt Weir
Editor
EMBO Reports

Referee #1:

This review article discusses the molecular mechanisms by which space flight and microgravity influence the skeletal muscle mass. The authors have provided good discussion about various aspects including the role of extracellular matrix components, sarcolemmal proteins, mechanosensitive ion channels, and cytoskeletal components in the induction of proteolytic systems and muscle atrophy in conditions of unloading and microgravity. While it is a well written review manuscript, there are couple of additional points which authors might want to include in this manuscript.

1) The authors have mentioned the activation of proteolytic system and FOXOs in various models of unloading/microgravity. However, it would be nice to discuss some signaling pathways that are activated and impinge on the proteolytic systems. For example, earlier studies from Susan Kandarian's group showed that unloading activates NF-kappa B signaling in mouse models of unloading (PMID: 15546001). There are some other pathways that might be affected.

2) Similarly, authors have mentioned that dystrophin-glycoprotein complex (DGC) is a signaling hub in sarcolemma. It would be informative to include some of the published studies on how disruption of this complex alters the activation of specific signaling pathways which also regulate the activation of UPS and autophagy (e.g., PubMed ID: PMID: 14519659; 12221078; 14718391)

3) Similarly, the role of mechanosensitive ion channels in the regulation of downstream signaling pathway in addition to calcium signaling should be discussed.

Referee #2:

This manuscript provides a concise and clearly written overview of the potential mechanisms by which reduced mechanical loading may be sensed and translated into signaling events that promote skeletal muscle atrophy. The topic is important, and the authors outline several plausible mechanotransduction pathways that could contribute to disuse-induced muscle loss.

However, the review lacks depth and critical engagement with the recent literature. The discussions are largely descriptive and do not sufficiently evaluate the strength of the experimental evidence supporting the involvement of the proposed mechanisms. More detailed consideration of key studies published in recent years, particularly those that directly test causality in relevant in vivo or genetic models, would substantially strengthen the manuscript.

Overall, the article reads as a relatively general overview of mechanotransduction in skeletal muscle, a subject that has been extensively reviewed in the past several years (see examples below). In this reviewers opinion, the manuscript does not provide the level of synthesis, conceptual advancement, or new perspective that would be expected for publication in EMBO Reports. A more integrative framework that highlights unresolved questions, reconciles conflicting findings, or proposes a refined model of disuse sensing would enhance its impact and suitability for this venue.

Examples of topically similar reviews

Chatzinikita, E., Maridaki, M., Philippou, A. Mechanotransduction in Skeletal Muscle. 2025. In: Apostolopoulos, N.C., Bogdanis, G.C., Seagrave, L.R., Plyley, M.J. (eds) Fundamentals of Recovery, Regeneration, and Adaptation to Exercise Stress: An Integrated Approach. Springer, Cham.

Joshi AS, Tomaz da Silva M, Kumar S, Kumar A. Signaling networks governing skeletal muscle growth, atrophy, and cachexia. Skelet Muscle. 2025 Oct 10;15(1):29. doi: 10.1186/s13395-025-00397-z. PMID: 41074035; PMCID: PMC12512606.

Wang Y, Meng J, Zhang J, Tian L, Wei W, Tang X, Zhang Q, Ding D, Wang X, Guo Z, He Y. Cell biomechanics on muscle atrophy: from intricate mechanisms to therapeutic frontiers. Ann Med. 2025 Dec;57(1):2540598. doi: 10.1080/07853890.2025.2540598. Epub 2025 Aug 1. PMID: 40747836; PMCID: PMC12320261.

Greyvenstein D, Newbold JP, Phillips SM. Tension to Translation: External to Internal Processes in Muscle Hypertrophy. Physiology (Bethesda). 2025 Dec 1. doi: 10.1152/physiol.00034.2025. Epub ahead of print. PMID: 41324917.

Mohamed JS, Pardo PS, Boriek AM. Mechano-Signal Transduction Pathways of the Diaphragmatic Muscle and Role of Cytoskeleton. Genes (Basel). 2025 Aug 18;16(8):968. doi: 10.3390/genes16080968. PMID: 40870015; PMCID: PMC12385653.

Kamal KY, Trombetta-Lima M. Mechanotransduction and Skeletal Muscle Atrophy: The Interplay Between Focal Adhesions and Oxidative Stress. Int J Mol Sci. 2025 Mar 20;26(6):2802. doi: 10.3390/ijms26062802. PMID: 40141444; PMCID: PMC11943188.

Response to reviewers comments

Referee #1:

This review article discusses the molecular mechanisms by which space flight and microgravity influence the skeletal muscle mass. The authors have provided good discussion about various aspects including the role of extracellular matrix components, sarcolemmal proteins, mechanosensitive ion channels, and cytoskeletal components in the induction of proteolytic systems and muscle atrophy in conditions of unloading and microgravity. While it is a well written review manuscript, there are couple of additional points which authors might want to include in this manuscript.

We thank the reviewer for the positive assessment of our manuscript and for the helpful suggestions. We have revised the manuscript accordingly.

1) The authors have mentioned the activation of proteolytic system and FOXOs in various models of unloading/microgravity. However, it would be nice to discuss some signaling pathways that are activated and impinge on the proteolytic systems. For example, earlier studies from Susan Kandarian's group showed that unloading activates NF-kappa B signaling in mouse models of unloading (PMID: 15546001). There are some other pathways that might be affected.

We thank the reviewer for this important suggestion. We have expanded the section on signaling pathways that impinge on proteolytic systems during unloading. Specifically, we now include a discussion of NF-κB signaling based on seminal work from Kandarian and colleagues, describing its activation in unloading models and its contribution to the induction of muscle-specific ubiquitin ligases. We also discuss how NF-κB may act in parallel or cooperatively with FOXO pathways to regulate atrophy-related gene expression (see revised section "Cellular responses to reduced mechanical loading").

2) Similarly, authors have mentioned that dystrophin-glycoprotein complex (DGC) is a signaling hub in sarcolemma. It would be informative to include some of the published studies on how disruption of this complex alters the activation of specific signaling pathways which also regulate the activation of UPS and autophagy (e.g., PubMed ID: PMID: 14519659; 12221078; 14718391)

We agree and have expanded the discussion of the dystrophin–glycoprotein complex (DGC) as a signaling hub. The revised manuscript now includes additional studies demonstrating how disruption of DGC integrity alters mechanotransduction and activates stress-responsive

pathways, including MAPK, NF-κB, and calcium-dependent signaling. We further discuss how DGC remodeling may impair insulin/PI3K-AKT signaling and sensitize muscle to proteolytic activation (see section “The dystrophin-glycoprotein complex contributes to integrin signaling”).

3) Similarly, the role of mechanosensitive ion channels in the regulation of downstream signaling pathway in addition to calcium signaling should be discussed.

We have substantially expanded this section to highlight broader roles of mechanosensitive ion channels beyond calcium influx. Specifically, we now discuss how channels such as Piezo1 can regulate transcriptional programs (e.g., YAP/TAZ signaling), mitochondrial function, reactive oxygen species production, and kinase signaling pathways. These additions emphasize the role of ion channels as integrators of mechanical and biochemical signals rather than simple conduits for calcium entry (see section “Mechanosensitive ion channels as modulators of unloading responses”).

Referee #2:

This manuscript provides a concise and clearly written overview of the potential mechanisms by which reduced mechanical loading may be sensed and translated into signaling events that promote skeletal muscle atrophy. The topic is important, and the authors outline several plausible mechanotransduction pathways that could contribute to disuse-induced muscle loss.

We thank the reviewer for the thoughtful and constructive critique. We have substantially revised the manuscript to address these concerns by increasing depth, incorporating recent literature, and strengthening the conceptual framework.

However, the review lacks depth and critical engagement with the recent literature.

We appreciate this comment and have revised the manuscript to provide a more critical and integrative analysis of the literature. We now explicitly distinguish between correlative observations and studies that test causality, particularly in the context of PI3K-AKT-FOXO signaling, mechanosensitive ion channels, and cytoskeletal remodeling. We also incorporated additional recent studies where relevant and clarified the limitations of current experimental approaches, especially regarding *in vivo* mechanistic evidence (see revised sections throughout, particularly “Cellular responses to reduced mechanical loading”).

The discussions are largely descriptive and do not sufficiently evaluate the strength of the experimental evidence supporting the involvement of the proposed mechanisms.

To address this point, we have introduced a unifying conceptual framework in both the Introduction and Conclusions. We propose that disuse atrophy emerges from a **distributed mechanosensory network**, rather than a single sensor, in which membrane tension, adhesion

complexes, cytoskeletal organization, and ion channel activity act as parallel inputs converging on shared catabolic pathways. This framework distinguishes **primary mechanosensing events** from **secondary adaptive responses**, and emphasizes their temporal coordination. We believe this provides a more integrative perspective that advances current models of disuse sensing.

More detailed consideration of key studies published in recent years, particularly those that directly test causality in relevant in vivo or genetic models, would substantially strengthen the manuscript.

We agree and have addressed this by explicitly discussing the limitations of current evidence. We now highlight that many conclusions in the field are based on correlative data and that relatively few studies directly establish causality *in vivo* under unloading conditions. We include specific examples (e.g., partial rescue of atrophy by AKT activation, limitations of *in vitro* models) and emphasize the need for time-resolved and genetically controlled studies to define primary mechanosensing mechanisms (see revised sections “Cellular responses to reduced mechanical loading” and “Conclusions”).

Overall, the article reads as a relatively general overview of mechanotransduction in skeletal muscle, a subject that has been extensively reviewed in the past several years (see examples below). In this reviewers opinion, the manuscript does not provide the level of synthesis, conceptual advancement, or new perspective that would be expected for publication in EMBO Reports. A more integrative framework that highlights unresolved questions, reconciles conflicting findings, or proposes a refined model of disuse sensing would enhance its impact and suitability for this venue.

We thank the reviewer for this comment. To address this concern, we have strengthened the manuscript in three ways:

1. **Conceptual integration**: introducing the mechanosensory network model as a unifying framework
2. **Critical evaluation**: distinguishing causality from correlation across pathways
3. **Future directions**: adding a dedicated section (“Box 1: In need of answers”) that highlights key unresolved questions and experimental challenges

We believe these additions provide a clearer synthesis and a forward-looking perspective that distinguishes this review from existing literature.

Examples of topically similar reviews

Chatzinikita, E., Maridaki, M., Philippou, A. Mechanotransduction in Skeletal Muscle. 2025. In: Apostolopoulos, N.C., Bogdanis, G.C., Seagrave, L.R., Plyley, M.J. (eds) Fundamentals of Recovery, Regeneration, and Adaptation to Exercise Stress: An Integrated Approach. Springer, Cham.

Joshi AS, Tomaz da Silva M, Kumar S, Kumar A. Signaling networks governing skeletal muscle growth, atrophy, and cachexia. Skelet Muscle. 2025 Oct 10;15(1):29. doi: 10.1186/s13395-025-00397-z. PMID: 41074035; PMCID: PMC12512606.

Wang Y, Meng J, Zhang J, Tian L, Wei W, Tang X, Zhang Q, Ding D, Wang X, Guo Z, He Y. Cell biomechanics on muscle atrophy: from intricate mechanisms to therapeutic frontiers. Ann Med. 2025 Dec;57(1):2540598. doi: 10.1080/07853890.2025.2540598. Epub 2025 Aug 1. PMID: 40747836; PMCID: PMC12320261.

Greyvenstein D, Newbold JP, Phillips SM. Tension to Translation: External to Internal Processes in Muscle Hypertrophy. Physiology (Bethesda). 2025 Dec 1. doi: 10.1152/physiol.00034.2025. Epub ahead of print. PMID: 41324917.

Mohamed JS, Pardo PS, Boriek AM. Mechano-Signal Transduction Pathways of the Diaphragmatic Muscle and Role of Cytoskeleton. Genes (Basel). 2025 Aug 18;16(8):968. doi: 10.3390/genes16080968. PMID: 40870015; PMCID: PMC12385653.

Kamal KY, Trombetta-Lima M. Mechanotransduction and Skeletal Muscle Atrophy: The Interplay Between Focal Adhesions and Oxidative Stress. Int J Mol Sci. 2025 Mar 20;26(6):2802. doi: 10.3390/ijms26062802. PMID: 40141444; PMCID: PMC11943188.

Prof. Shenhav Shemer
Technion Institute of Technology
Biology
Faculty of Biology, Room 214, Technion city
Haifa, - 3200003
Israel

Dear Prof. Shemer,

I am pleased to inform you that your manuscript has been accepted for publication in EMBO reports. Your manuscript will be processed for publication by EMBO Press. It will be copy edited and you will receive page proofs prior to publication. Please note that you will be contacted by Springer Nature Author Services to complete licensing information.

Should you be planning a Press Release on your article, please notify the production team via this form in order to coordinate publication and release dates: https://forms.cloud.microsoft/e/eH4A8JVP6g

If you have any questions, please do not hesitate to contact the Editorial Office. Thank you for your contribution to EMBO Reports.

Yours sincerely,

Kurt Weir
Editor
EMBO Reports

>>> Please note that it is EMBO Reports policy for the transcript of the editorial process (containing referee reports and your response letter) to be published as an online supplement to each paper. If you do NOT want this, you will need to inform the Editorial Office via email immediately. More information is available here: https://link.springer.com/partners/embo-press/editorial-policies#Peer%20review
